# Use of next-generation sequencing to detect *LDLR* gene copy number variation in familial hypercholesterolemia[S]

**DOI:** 10.1194/jlr.D079301

**Published:** 2017-09-05

**Authors:** Michael A. Iacocca, Jian Wang, Jacqueline S. Dron, John F. Robinson, Adam D. McIntyre, Henian Cao, Robert A. Hegele

**Affiliations:** Departments of Medicine and Biochemistry, Schulich School of Medicine & Dentistry,* Western University, London, Ontario, Canada; Robarts Research Institute,† Western University, London, Ontario, Canada

**Keywords:** diagnostic tools, lipid and lipoprotein metabolism, molecular biology/genetics, LDL, lipoprotein receptors, coronary heart disease, precision medicine, DNA variation, genetic testing, bioinformatics

## Abstract

Familial hypercholesterolemia (FH) is a heritable condition of severely elevated LDL cholesterol, caused predominantly by autosomal codominant mutations in the LDL receptor gene (*LDLR*). In providing a molecular diagnosis for FH, the current procedure often includes targeted next-generation sequencing (NGS) panels for the detection of small-scale DNA variants, followed by multiplex ligation-dependent probe amplification (MLPA) in *LDLR* for the detection of whole-exon copy number variants (CNVs). The latter is essential because ∼10% of FH cases are attributed to CNVs in *LDLR*; accounting for them decreases false negative findings. Here, we determined the potential of replacing MLPA with bioinformatic analysis applied to NGS data, which uses depth-of-coverage analysis as its principal method to identify whole-exon CNV events. In analysis of 388 FH patient samples, there was 100% concordance in *LDLR* CNV detection between these two methods: 38 reported CNVs identified by MLPA were also successfully detected by our NGS method, while 350 samples negative for CNVs by MLPA were also negative by NGS. This result suggests that MLPA can be removed from the routine diagnostic screening for FH, significantly reducing associated costs, resources, and analysis time, while promoting more widespread assessment of this important class of mutations across diagnostic laboratories.

Familial hypercholesterolemia (FH) is an inherited dyslipidemia characterized by a lifelong exposure to elevated LDL cholesterol levels with increased risk of premature atherosclerosis causing CVD, particularly coronary heart disease (CHD) (1). Although FH has multiple genetic etiologies, ∼90% of molecularly defined cases result from autosomal codominant mutations in the LDL receptor gene (*LDLR*) (1, 2). Pathogenic loss-of-function variants in *LDLR* affect every functional domain of the encoded protein and include a spectrum of both point mutations and large-scale deletions or duplications spanning whole exons—known as copy number variants (CNVs) (3). Autosomal codominant FH may also occasionally be caused by specific protein-altering mutations in the apo B gene (*APOB*) or by gain-of-function mutations in the proprotein convertase subtilisin/kexin type 9 gene (*PCSK9*). Heterozygous FH (HeFH) has been shown to be more common than previously thought: recent population-based studies in the United Kingdom (4), the Netherlands (5), Northern Europe (6), Poland (7), and the United States (8) suggest that HeFH affects ∼1 in 250 individuals in the general population. Furthermore, the prevalence in certain founder populations is even higher, such as ∼1 in 200 in French Canadians, ∼1 in 165 in Tunisians, ∼1 in 85 in Christian Lebanese, and ∼1 in 72 in South African Afrikaners (9). Homozygous FH (HoFH) is rare, with an incidence of ∼1 in 160,000 to 1 in 300,000 (5). By using these prevalence figures, there are ∼34 million individuals globally with FH; however, <1% have been diagnosed, although detection rates vary widely by country (10). Early identification and treatment of FH patients is essential, as lipid-lowering therapies have been proven to reduce LDL cholesterol levels to those of the general population (1).

Recently, FH has progressed toward the forefront of precision medicine as patients worldwide are more commonly offered genetic testing in diagnosis (1, 11). Advantages of genetic testing for FH are manifold. They include: *1*) achieving certainty in the context of incomplete clinical criteria, such as family history or typical physical findings; *2*) motivating cascade screening of family members; *3*) initiating genotype-directed treatment strategies (12); and *4*) supporting insurance coverage of certain medications. Successful molecular diagnosis depends on the ability of the designated method to assess both locus and allele heterogeneity associated with FH (11). The cost-effectiveness of such methods may limit their widespread implementation and routine use. Traditionally, the genetic screening strategy for FH has been Sanger sequencing for assessment of all coding regions in *LDLR* plus one or two specific exons in *APOB*, followed by multiplex ligation-dependent probe amplification (MLPA) for detection of CNVs in *LDLR* (13). The latter method is essential as ∼10% of FH cases have been attributed to CNVs in *LDLR* (13); identifying CNVs increases diagnostic yield, avoiding false-negative diagnoses. Next-generation sequencing (NGS) techniques offer superior analysis with the potential to assess a wider range of genetic abnormalities. However, the ability to detect CNVs in *LDLR* using NGS data outputs is currently unevaluated. Accurate identification of CNV mutations from NGS data is important because this class of variation comprises a significant proportion of FH cases and not all sequencing facilities have the resources, time, or interest to establish a parallel MLPA system for detecting them. We have developed a dedicated high-coverage targeted NGS panel to detect rare variants in several dyslipidemias, of which FH is the most important clinically (14). Here, we tested whether NGS data could be bioinformatically probed to detect CNVs in the *LDLR* gene and compared the results with MLPA, which is currently considered to be the “reference standard” or “gold standard” method to detect such variants.

## METHODS

### Study subjects

We studied 388 Canadian individuals aged ≥18 years who were referred to a tertiary lipid clinic for treatment of severe hypercholesterolemia. Diagnosis of at least possible FH was made by using the Dutch Lipid Clinic Network criteria; all patients had untreated LDL cholesterol ≥5 mmol/l (194 mg/dl), plus family history of hypercholesterolemia, plus some with either personal or family history of premature CHD. Our protocol was approved by the Western University Research Ethics Board, and all participants provided informed consent for genetic analyses. A total of 313 patients studied here were part of our recent report on polygenic FH (15).

### Targeted NGS

Genomic DNA was isolated from whole blood by using the Puregene DNA Blood Kit (Gentra Systems, Qiagen, Mississauga, Canada) and was subject to targeted NGS using our LipidSeq panel (14). With LipidSeq, each sample is sequenced for 73 key genes in lipid metabolism, including all coding regions, ∼150 bp at intron-exon boundaries, and ∼1,000 bp of the 5′ untranslated region (UTR) of all FH major and minor phenocopy genes, namely, *LDLR*, *APOB*, *PCSK9*, *LDLRAP1*, *APOE*, *STAP1*, *LIPA*, *ABCG5*, and *ABCG8*. Library preparation was performed by using the Nextera Rapid Capture Custom Enrichment kit (Illumina, San Diego, CA), and enriched samples were sequenced on a MiSeq personal sequencer platform (Illumina) by using 2× 150-bp paired-end chemistry and in accordance with manufacturer instructions. MiSeq-generated .FASTQ files were downloaded and processed individually by using a custom automated workflow in CLC Genomics Workbench version 8.51 (CLC Bio, Aarhus, Denmark) for sequence alignment (mapped to human genome build GRCh37/hg19), variant calling (generation of .VCF files), and target region coverage statistics (generation of .BAM files). Variant annotation was conducted by using ANNOVAR (www.qiagenbioinformatics.com/products/annovar) with a customized script. Our LipidSeq method has an average depth of coverage (DOC) of 300-fold for each base. Attribution of pathogenicity for detected variants was performed as previously described (15, 16). Sanger sequencing was used to confirm the presence of small-scale pathogenic variants detected by LipidSeq.

### CNV detection by MLPA

The MLPA Salsa P062-D2 kit (MRC Holland, Amsterdam, The Netherlands) was used for the detection of large-scale whole-exon deletion and duplication events in *LDLR*. The P062-D2 kit contains 20 probes for *LDLR* (one for each of the promoter and all 18 exons, with the exception of two for exon 15), plus one flanking probe for upstream of *LDLR* and 12 reference probes for gene loci on alternative autosomal chromosomes. The probe mix also contains nine control fragments that generate short products to indicate that the DNA quantity and ligation reaction are sufficient for proper analysis. The principles and stages of probe hybridization are as previously described (17), and protocol followed the manufacturer’s guide version MDP-005 (www.mrc-holland.com). PCR amplification was carried out in a Veriti thermocycler (Applied Biosystems, Foster City, CA), and products were subsequently analyzed by using a 3730 Automated DNA Sequencer (Applied Biosystems). MLPA fragment analysis was performed by using Coffalyser software version 140721.1958 (MRC Holland; www.coffalyser.net), where relative amounts of probe-amplified products are compared with normal controls (samples within the same run) to determine the copy number state for each target region. We used one normal control sample per seven study samples. Ratio values <0.75 indicating copy number loss and >1.33 indicating copy number gain were flagged. Two-sample *t*-tests were used for all statistical comparisons against the profiles of normal controls (*P* < 0.05).

### CNV detection by NGS data

The bioinformatics tool CNV Caller, an application within the variant annotation software VarSeq v1.4.3 (Golden Helix, Bozeman, MT), was used for analysis of our existing LipidSeq data set for CNV detection. VarSeq CNV Caller requires .VCF and .BAM files (generated by NGS; see above) as inputs for each sample, plus a .BED file that defines the target region chromosomal and probe start/stop coordinates for the specific NGS panel used. Briefly, the VarSeq algorithm uses normalized DOC analysis as its principal method, whereby an increase in sample DOC across a target region, when compared with reference controls, suggests a gain in genomic material, and a decrease in sample DOC suggests a loss.

To first normalize the raw coverage data, the VarSeq algorithm uses a set of matched reference controls. We provided the algorithm a population of >100 normal controls, from which it selected 30 with the lowest percent difference in coverage data compared with the sample of interest; samples were flagged if the average percent difference was >20%. Matched reference controls were further used to correct for GC-content bias and regions that were relatively unamenable to mapping. A ratio and z-score metric were then computed for each target region. The ratio was calculated as the sample coverage divided by the mean reference sample coverage. The z-score measured the number of SDs that a sample’s coverage was from the mean reference sample coverage. A Bayesian frame network model then assigned CNV state based on the probability that for each target region these two metrics represent a: *1*) diploid (normal) state; *2*) heterozygous deletion; *3*) homozygous deletion; or *4*) duplication event. Furthermore, the algorithm also exploited SNP heterozygosity information across a target region as an additional supporting metric for assigning CNV state. Denoted as variant allele frequencies (VAFs), a VAF of any non-0 or 1 value provided further evidence against deletions, whereas a VAF such as 1/3 or 2/3 provided further evidence for duplications. Finally, segmentation analysis merged multiple affected target regions to characterize contiguous CNV events; the minimum limit of CNV detection was the smallest whole-exon (lower limit ∼300 bp), whereas the maximum limit was the entire *LDLR* gene (approximately 18 kb).

### CNV filtration

After CNV analysis, CNVs were filtered based on mutually inclusive ratio and z-score thresholds. A ratio threshold value of ≤0.7 and z-score of ≤−5.0 were used to identify probable heterozygous deletions, whereas a ratio value of ≥1.30 and z-score of ≥5.0 were used for duplications. For further validation, evidence from target region VAFs were also manually evaluated as explained above. For the purposes of this study, only CNVs detected in *LDLR* were considered.

### Statistical analyses

Analyses of demographic features were performed in SAS version 9.1 (SAS Institute, Cary, NC). Quantitative traits were compared by using unpaired *t*-tests, while discrete traits were compared by using chi-square analysis, typically 2 × 2 contingency analyses. The nominal level of statistical significance was set at *P* < 0.05.

## RESULTS

### Study sample demographics

Baseline clinical and biochemical features of the individuals studied here are shown in **Table 1**.

**TABLE 1.** Patient demographics for Canadian FH cohort

	Overall (*N* = 388)	Women (*N* = 212)	Men (*N* = 176)
Age, years	50.7 ± 15.2	52.1 ± 16.3	48.9 ± 13.6
Body mass index, kg/m^2^	27.8 ± 5.9	27.3 ± 6.1	28.6 ± 5.5
Total cholesterol, mmol/l	8.94 ± 1.91	9.13 ± 1.94	8.66 ± 1.83
LDL cholesterol, mmol/l	6.79 ± 1.79	6.93 ± 1.80	6.60 ± 1.76
HDL cholesterol, mmol/l	1.35 ± 0.38	1.43 ± 0.39	1.22 ± 0.35
Triglyceride, mmol/l	1.79 ± 0.88	1.77 ± 0.98	1.81 ± 0.73
Personal history of CVD,*^a^* %	17.9	12.9	25.0
Family history of CVD,*^a^* %	40.0	44.7	50.0
Definite or probable FH (DLCN score),*^a^* %	65.5	67.1	63.3

Values are represented as mean ± SD. CVD indicates CVD onset <55 years in men and <60 years in women. DLCN, Dutch Lipid Clinic Network.

a Based on complete data from 145 individuals.

### CNVs detected by MLPA

**Fig. 1.**
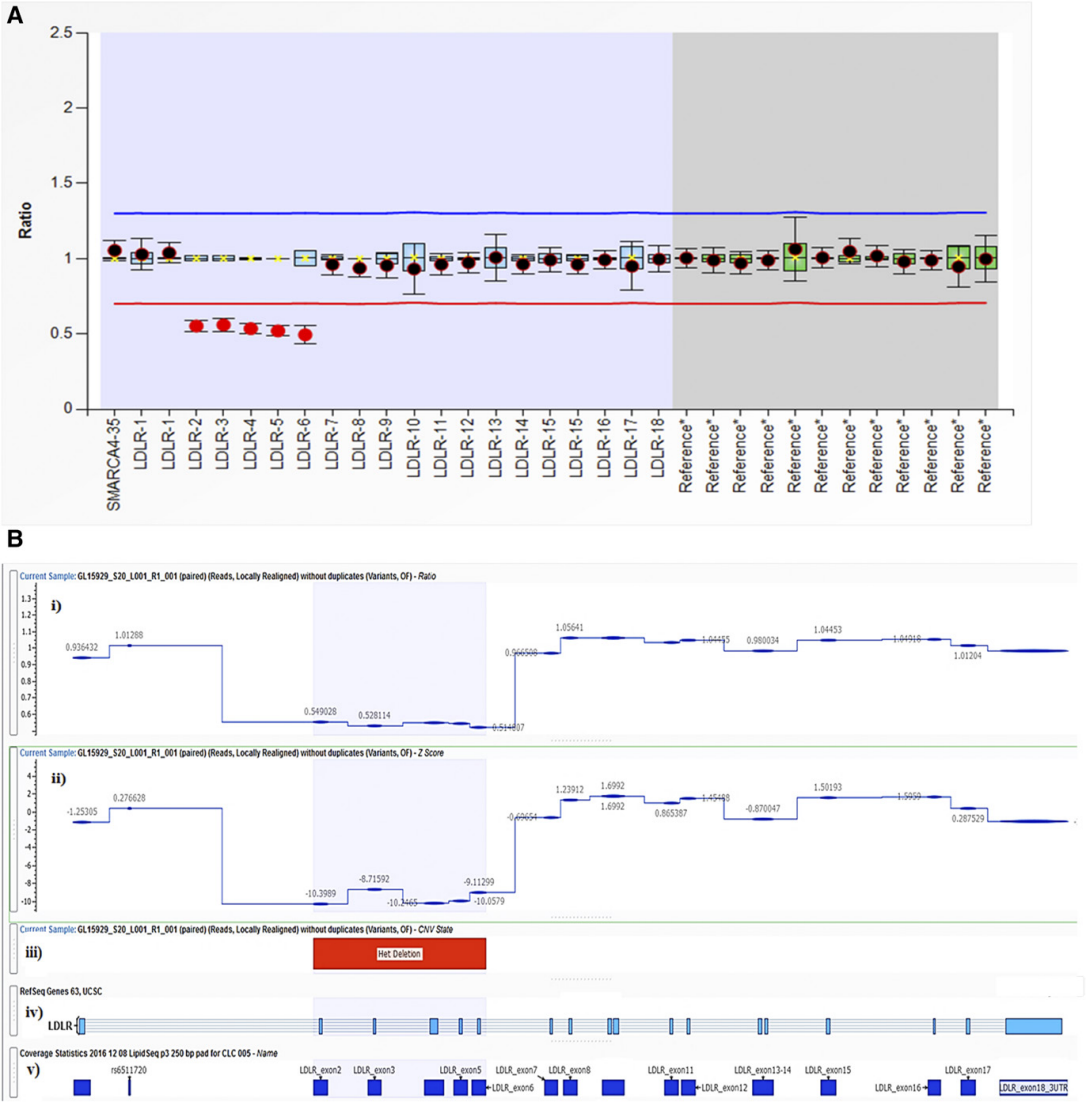
Two methods of detection of a CNV deletion event in the *LDLR* gene in a patient with FH (subject GL15929) with a heterozygous deletion in *LDLR* exons 2–6. A: MLPA method output: heterozygous deletion in *LDLR* exons 2–6. Exon numbers are shown by “LDLR-N” (where N is the number of the exon, the first “LDLR-1” indicates the promoter, and “SMARCA4-35” is upstream of the promoter), and “*Reference” indicate reference probes bound to alternative chromosomes. For each probe target region, two separate plots are generated: *1*) the normalized reference sample set is represented by 1-SD box plots, where “X” indicates the mean and the horizontal line the median probe-signal intensity; and *2*) the normalized patient sample probe-signal ratio is overlaid as a dot and is surrounded by error bars depicting the 95% CI. The upper arbitrary border (blue line) and lower arbitrary border (red line) are placed ±0.3 from the reference sample mean of each probe. B: VarSeq CNV Caller method output: heterozygous deletion in *LDLR* exons 2–6. Different regions of the output are as follows. i: Normalized ratio metric computed for each LipidSeq target region in *LDLR*; depth of sequence coverage comparative to reference controls, where ∼1.0 indicates diploid (normal) copy number state and ∼0.50 indicates a heterozygous deletion event. ii: Normalized z-score metric; number of SDs the DOC is from the reference control mean coverage, where ≤−5.0 is the threshold set to indicate a deletion event. iii: CNV state, determined by ratio and z-score metrics together with supporting evidence from VAFs (not shown). Segmentation analysis has merged multiple affected target regions to call a contiguous heterozygous deletion event. iv: Exon map of *LDLR* gene. v: LipidSeq probe target regions.

Thirty-eight (9.8%) of 388 FH patients were positive for whole-exon CNVs in *LDLR* detected by MLPA (**Table 2**). The majority (35 of 38; 92.1%) of these patients had heterozygous deletions, of which 13 spanned multiple exons. There were three detected duplications. The most common CNV involved a heterozygous deletion of the promoter and exon 1, found in 22 of 38 (57.9%) CNV-positive patients. Exon 6 was affected in 6 of 38 (15.8%) patients. Of the 18 exons in *LDLR*, only exons 8, 9, and 10 were unaffected by CNV events among the study sample. All control samples had normal MLPA profiles. Sample outputs from MLPA for two different types of CNVs are shown in **Figs. 1** and ** 2**.

**TABLE 2.** *LDLR* whole-exon CNVs identified in 388 patients with FH

Sample no.	MLPA		NGS data		
	Type	Region	Detection	Ratio	Z-score
GL133	Het. deletion	Promoter-exon 1	Yes	0.62	−6.1
GL474	Het. deletion	Promoter-exon 1	Yes	0.43	−9.8
GL2180	Het. deletion	Promoter-exon 1	Yes	0.58	−5.6
GL3185	Het. deletion	Promoter-exon 1	Yes	0.51	−6.2
GL3767	Het. deletion	Promoter-exon 1	Yes	0.50	−8.2
GL4120	Het. deletion	Promoter-exon 1	Yes	0.53	−7.9
GL4631	Het. deletion	Promoter-exon 1	Yes	0.52	−8.7
GL6406	Het. deletion	Promoter-exon 1	Yes	0.56	−8.7
GL8496	Het. deletion	Promoter-exon 1	Yes	0.51	−8.6
GL8874	Het. deletion	Promoter-exon 1	Yes	0.52	−7.9
GL9037	Het. deletion	Promoter-exon 1	Yes	0.51	−7.5
GL12366	Het. deletion	Promoter-exon 1	Yes	0.59	−6.6
GL12367	Het. deletion	Promoter-exon 1	Yes	0.55	−5.4
GL12533	Het. deletion	Promoter-exon 1	Yes	0.51	−6.2
GL14152	Het. deletion	Promoter-exon 1	Yes	0.59	−6.0
GL14549	Het. deletion	Promoter-exon 1	Yes	0.51	−8.0
GL15102	Het. deletion	Promoter-exon 1	Yes	0.59	−6.6
GL15358	Het. deletion	Promoter-exon 1	Yes	0.46	−8.7
GL15491	Het. deletion	Promoter-exon 1	Yes	0.51	−8.1
GL15561	Het. deletion	Promoter-exon 1	Yes	0.57	−6.2
GL15575	Het. deletion	Promoter-exon 1	Yes	0.51	−9.8
GL15992	Het. deletion	Promoter-exon 1	Yes	0.57	−6.8
GL14257	Het. deletion	Promoter-exon 2	Yes	0.57	−5.6
GL14258	Het. deletion	Promoter-exon 2	Yes	0.50	−9.5
GL5014	Het. deletion	Promoter-exon 6	Yes	0.54	−7.4
GL8531	Het. deletion	Exons 2–3	Yes	0.56	−6.7
GL15929	Het. deletion	Exons 2–6	Yes	0.54	−9.7
GL9910	Duplication	Exons 2–6	Yes	1.38	11.8
GL6260	Het. deletion	Exons 3–6	Yes	0.53	−9.7
GL15692	Het. deletion	Exons 5–6	Yes	0.54	−14.7
GL12812	Duplication	Exon 7	Yes	1.47	7.3
GL5843	Duplication	Exons 11–12	Yes	1.86	12.7
GL12450	Het. deletion	Exons 11–12	Yes	0.54	−7.8
GL15560	Het. deletion	Exons 13–14	Yes	0.52	−15.9
GL15110	Het. deletion	Exons 13–15	Yes	0.65	−8.7
GL5799	Het. deletion	Exons 16–18	Yes	0.53	−9.9
GL4789	Het. deletion	Exons 17–18	Yes	0.53	−9.3
GL2381	Het. deletion	Exons 17–18	Yes	0.55	−10.1

For multi-exon CNVs the reported ratio and z-score values are averaged across each affected region (for individual values see supplemental Table S1). Het., heterozygous.

**Fig. 2.**
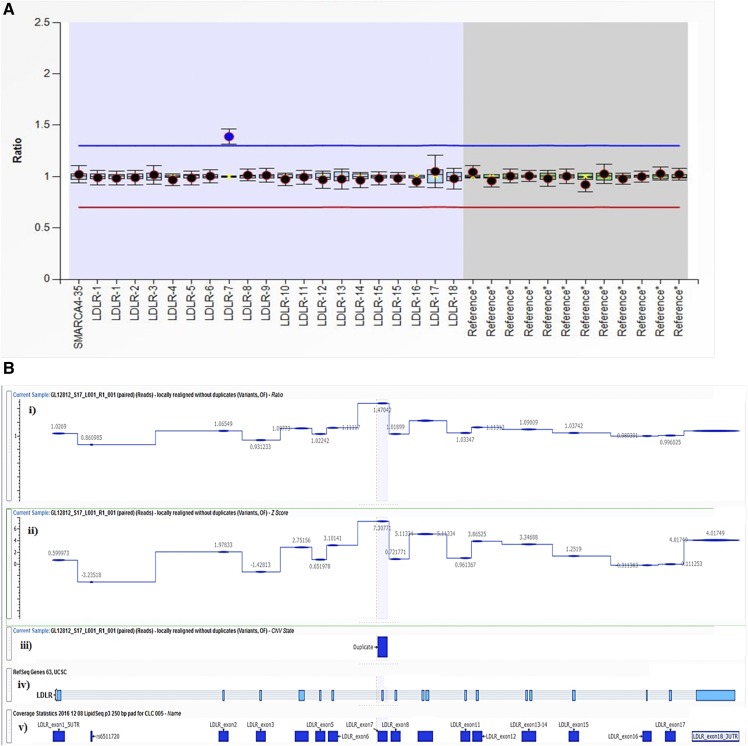
Two methods of detection of a CNV duplication event in the *LDLR* gene in a patient with FH (subject GL12812) with a duplication in *LDLR* exon 7. See Fig. 1 legend for overall structure of the panels. A: MLPA method output: duplication in *LDLR* exon 7. B: VarSeq CNV Caller method output: duplication in *LDLR* exon 7. i: Normalized ratio metric computed for each LipidSeq target region in *LDLR*; depth of sequence coverage comparative to reference controls where ∼1.0 indicates diploid (normal) copy number state and ∼1.5 indicates a duplication event. ii: Normalized z-score metric; number of SDs the DOC is from the reference control mean coverage, where ≥5.0 is the threshold set to indicate a duplication event; other sections are as in Fig. 1.

### CNVs detected by NGS data

Thirty-eight (9.8%) of 388 FH patients were positive for CNVs in *LDLR* detected by NGS. These CNVs and their associated states were in 100% concordance with those detected by MLPA (Table 2). Furthermore, the 350 samples negative for CNVs by MLPA were also negative by NGS. Using MLPA as the “gold standard,” there were no false positives and no false negatives using our bioinformatics procedure applied to NGS data, which translates to a diagnostic test specificity and sensitivity of 100% each (**Table 3**). Sample outputs from VarSeq CNV Caller for two different types of CNVs corresponding to MLPA tracings referred to above are shown in Figs. 1 and 2.

**TABLE 3.** A 2 × 2 contingency analysis of copy number variants called by MLPA and NGS methods

NGS data result	MLPA result	
	CNV	Diploid
Positive	True positives: 38	False positives: 0
Negative	False negatives: 0	True negatives: 350

Sensitivity: 100%; specificity: 100%.

## DISCUSSION

The principal new finding here is that NGS data for the *LDLR* gene that is processed bioinformatically from patient samples referred for assessment of FH has a 100% concordance rate for calling of large-scale CNVs using MLPA as the “gold standard” reference method. The ability to detect the full spectrum of mutations in *LDLR* is critical in obtaining a molecular diagnosis for FH, especially since up to 10% or more of such mutations are large-scale CNVs rather than small-scale DNA sequence variants, depending on the cohort and ascertainment (18). The current procedure for diagnostic laboratories often includes targeted NGS followed by MLPA. Our findings suggest that the information about potential CNVs also resides within NGS data and that MLPA is potentially dispensable, particularly for the *LDLR* gene. NGS with appropriate bioinformatics has the ability to identify both small- and large-scale variant detection within a single platform and single analytic procedure.

Specifically, in our analysis of 388 samples referred for FH diagnosis, 38 reported CNVs detected by MLPA were also successfully detected by NGS; no sample that was positive for a CNV by MLPA was missed by our bioinformatic approach. Importantly, with a specificity and sensitivity of 100%, there were no false-positive or false-negative calls derived from NGS data compared with MLPA. Furthermore, this targeted NGS method identified a wide range of CNV events, including those affecting almost all 18 exons, both single- and multi-exon events, and both deletions and duplications (see Table 2).

The prevalence of whole-exon CNVs (9.8%) in FH patients is similar in our cohort compared with those previously studied (19, 20). The *LDLR* locus is known to have an especially high frequency of *Alu* repeat elements, making it susceptible to CNV mutagenesis by unequal homologous recombination events (19, 21). The pattern of CNV events detected across *LDLR* largely correlates with the distribution of these *Alu* repeats; sequence analysis in *LDLR* has revealed that the large majority of known CNV breakpoints are found within introns 1-8 and 12-3′ UTR, which is where *Alu* elements are most concentrated (22). This feature might explain why exons 8, 9, and 10 were unaffected by CNVs in our cohort. The high frequency of promoter-exon 1 heterozygous deletions can be attributed to the presence of French Canadians in our study sample. This ∼15 kb deletion is a well-known founder-effect mutation first discovered in 1987 to be present in 63% of French Canadians with FH, presumably originating among the 8,000 ancestors of the present-day French Canadians who have traditionally had little cross-breeding with other ethnic groups (23). Because of the high prevalence of this specific mutation, CNV analysis has long been an important component of FH screening in Canada.

For the last decade or more, MLPA has been regarded as the “gold standard” for CNV detection in *LDLR.* Prediction of CNVs from NGS data has been investigated previously; however, it remains a relatively new and challenging field. Commonly used CNV prediction programs include CoNIFER (24), ExomeDepth (25), ExomeCopy (26), XHMM (27), and CNV-seq (28); however, many of these designated methods have shown high rates of false-positive CNVs, which poses a major limitation on potential clinical use. Moreover, many of the literature-reported CNV prediction programs have been designed and optimized for whole-genome or whole-exome NGS analysis, which is inherently different from targeted NGS analysis, because the latter focuses on only a few target genes, with known reference copy number counts, and provides a higher average sequence coverage per base, which, in turn, allows for DOC methods to be a suitable approach. The higher DOC for each particular *LDLR* base using targeted NGS versus whole-genome or whole-exome NGS potentially increases the sensitivity to detect CNVs. Finally, our study took advantage of our unique large cohort of known *LDLR* MLPA positive and negative samples as reference standards to evaluate the applicability of this bioinformatics approach to CNV detection in the clinical diagnostic context for FH.

Essential to the performance of DOC analysis is use of appropriate matched reference controls for cross-sample normalization and comparison (i.e., controls sequenced with the exact enrichment chemistry and NGS panel version design as the sample of interest) and quality-control thresholds set for ratio and z-score metric outputs. Although proven robust in detection, our methodology has some limitations in further defining CNVs. In the event of a called “duplication,” the VarSeq CNV Caller output does not specify the exact degree of amplification. By design, this feature is a result of the difficulty in accurately differentiating DOC metrics as copy numbers incrementally increase. Another limitation is the inability to determine whole-exon CNV breakpoints as these reside in the intronic regions, which are unsequenced on our LipidSeq panel. Importantly, however, although such information may be useful for research purposes, it does not affect the documentation of a CNV for the purpose of diagnosis.

Transitioning to CNV detection from targeted NGS data has many benefits. Our cost for MLPA analysis in *LDLR*—including reagents, controls, duplicate analyses, and labor—was approximately $80 USD per patient sample, which totaled approximately $31,000 USD for this cohort of 388 FH individuals. These costs would be essentially eliminated when applying a bioinformatics method to NGS data as such data are already being generated for small-scale variant analysis that precedes CNV assessment. We have found that, once established, the bioinformatics workflow for CNV detection takes only an additional ∼10 min for a set of 24 samples. Furthermore, all targeted genes on the designated NGS panel are analyzed for CNVs concurrently; thus, CNV analysis can be extended to all FH-associated genes (in the case of LipidSeq), namely, *APOB*, *PCSK9*, *LDLRAP1*, *APOE*, *STAP1*, *LIPA*, *ABCG5*, and *ABCG8* at no extra cost. Although causative CNVs in these genes are expected to be rare, they have long remained uninvestigated because MLPA methods are either not available or not applied for genes outside the *LDLR*. Extending CNV analysis to all such FH-associated genes furthers our ability to account for all genetic abnormalities capable of explaining FH cases; this, in turn, further decreases false-negative findings. For instance, with this extended ability, we have now identified one patient with a whole-gene duplication in *APOB* and two patients with whole-gene duplications in *PCSK9* who otherwise had no mutations to explain their phenotype (data not shown). In conclusion, we report 100% concordance for the detection of whole-exon CNVs in *LDLR* between a bioinformatics approach applied to existing NGS data and the “gold standard” reference method of MLPA. This result suggests that the latter independent bench method can be removed from the routine molecular diagnostic workup for FH, improving costs, resources, and analysis time and thus encouraging an even more commonplace assessment of this important class of mutations across diagnostic laboratories in the future.
